# Rosuvastatin Improves Neurite Outgrowth of Cortical Neurons against Oxygen-Glucose Deprivation via Notch1-mediated Mitochondrial Biogenesis and Functional Improvement

**DOI:** 10.3389/fncel.2018.00006

**Published:** 2018-01-17

**Authors:** Weiliang He, Yingping Liu, Xiaochao Tian

**Affiliations:** ^1^Department of Neurology, Hebei General Hospital, Shijiazhuang, China; ^2^Department of Cardiology, Beijing Shijitan Hospital, Capital Medical University, Beijing, China; ^3^Department of Cardiology, The Second Hospital of Hebei Medical University, Shijiazhuang, China

**Keywords:** cortical neurons, neurite outgrowth, cerebral ischemia, rosuvastatin, mitochondria, notch1

## Abstract

Neurogenesis, especially neurite outgrowth is an essential element of neuroplasticity after cerebral ischemic injury. Mitochondria may supply ATP to power fundamental developmental processes including neuroplasticity. Although rosuvastatin (RSV) displays a potential protective effect against cerebral ischemia, it remains unknown whether it modulates mitochondrial biogenesis and function during neurite outgrowth. Here, the oxygen-glucose deprivation (OGD) model was used to induce ischemic injury. We demonstrate that RSV treatment significantly increases neurite outgrowth in cortical neurons after OGD-induced damage. Moreover, we show that RSV reduces the generation of reactive oxygen species (ROS), protects mitochondrial function, and elevates the ATP levels in cortical neurons injured by OGD. In addition, we found that, under these conditions, RSV treatment increases the mitochondrial DNA (mtDNA) content and the mRNA levels of mitochondrial transcription factor A (TFAM) and nuclear respiratory factor 1 (NRF-1). Furthermore, blocking Notch1, which is expressed in primary cortical neurons, reverses the RSV-dependent induction of mitochondrial biogenesis and function under OGD conditions. Collectively, these results suggest that RSV could restore neurite outgrowth in cortical neurons damaged by OGD *in vitro*, by preserving mitochondrial function and improving mitochondrial biogenesis, possibly through the Notch1 pathway.

## Introduction

Stroke is one of the leading causes of long-term disability and death worldwide and affects the patients’ emotional, mental and physical health (Thampy and Pais, 2016). Neurogenesis including the regulation of neurite outgrowth is believed to be vital as a mechanism of neuroplasticity after cerebral ischemic injury (Kitamura et al., 2009; Lin and Sheng, 2015). Therefore, targeting neurite outgrowth represents a prospective therapeutic strategy for stroke patients.

Neurite outgrowth is a developmental process that requires a heavy energy supply, provided by the mitochondria (Mattson and Partin, 1999). However, the disruption of oxygen and glucose supply, which is caused by stroke and mimicked by the *in vitro* oxygen-glucose deprivation (OGD) model, can produce a large number of reactive oxygen species (ROS) and lead to the depletion of cellular ATP (Rousset et al., 2015). Mitochondria are highly dynamic organelles and continually undergo biogenesis, fission and fusion (Anne Stetler et al., 2013). Maintaining a proper mitochondrial function depends on correct mitochondrial biogenesis (Sbert-Roig et al., 2016). Numerous studies have shown that mitochondrial dysfunction, especially regarding biogenesis, plays a crucial role in ischemic injury (McLeod et al., 2005; Gutsaeva et al., 2008). Therefore, identifying pharmacological agents that preserve mitochondrial functions and promote neurite outgrowth against cerebral ischemic injury might be an ideal therapeutic strategy.

Statins are structural analogs of the 3-hydroxy-3-methylglutaryl coenzyme A (HMG-CoA), the substrate of HMG-CoA reductase, and have been used as potent cholesterol-lowering drugs for the treatment of hypercholesterolaemia and coronary heart disease (Stein, 2002; Rader, 2003). Many studies have shown that statins reduce stroke incidence and improve its outcome (Bösel et al., 2005). The role of statins in neurite outgrowth has been proposed by previous studies (Jin et al., 2012; Métais et al., 2015). Rosuvastatin (RSV) is considered as one of the most effective statins and is able to ability to form multiple polar covalent bonds with the HMG-CoA reductase. In mice, RSV was shown to have a neuroprotective effect following cerebral ischemia. Accumulating evidence suggests that statins decrease the oxidative phosphorylation capacity and membrane potential of mitochondria, thus impairing their function (Broniarek and Jarmuszkiewicz, 2016), However, a recent study reported that, in the kidneys of wild type C57BL/6 male mice, RSV increases the protein levels of Sirt1 and PGC-1a, two key players in mitochondrial biogenesis (Corsetti et al., 2014). Nevertheless, it is not completely clear whether RSV modulates mitochondrial function and biogenesis during neurite outgrowth.

The Notch pathway constitutes one of the most well-conserved developmental pathways throughout evolution. It controls both cell proliferation and apoptosis and is crucial for intercellular interactions in human development as well as in disease (Artavanis-Tsakonas et al., 1999; Bi and Kuang, 2015). A lot of attention has been paid to understanding how this pathway regulates cellular metabolism. Notch1, a well-studied protein, plays a significant role in this pathway (Kageyama et al., 2007). Recent studies have shown that the Notch1 pathway regulates mitochondrial fusion (Kasahara et al., 2013), but also affects their function (Basak et al., 2014), indicating that Notch1 is crucial in mitochondrial metabolism. Furthermore, the activation of Notch1 modulates the expression of important mitochondria-localized metabolic pathway proteins (Basak et al., 2014). Additionally, a previous study reported that Notch1 may exert a negative effect on neurite outgrowth (Berezovska et al., 1999). However, whether Notch1 mediates the protective effect of RSV on neurite outgrowth following ischemic injury is still not clear.

Based on this evidence, this study was designed to investigate the role of mitochondrial function and biogenesis in RSV-induced neurite outgrowth. We also attempted to determine the role of Notch1 in promoting the effects of RSV, in order to further elucidate the potential mechanism of its action.

## Materials and Methods

The experimental protocols were conducted in accordance with guidelines approved by the Animal Experimentation Ethics Committee of Hebei Medical University.

### Primary Cortical Neuron Culture and OGD

Cortical neurons were obtained from the brains of embryonic day 15–18 (E15–18) C57BL/6 mice (Vital River Laboratory Animal Technology Co. Ltd., Beijing, China). The cerebral cortex was dissected and incubated at 37°C, for 15 min, in Hibernate-E solution (Sigma, Ronkonkoma, NY, USA), supplemented with papain (2.0 mg/ml, Sigma, Ronkonkoma, NY, USA). Then, the cortical tissues were neutralized and dissociated into single cells in Neurobasal medium containing 2% B-27 supplement (Invitrogen, Carlsbad, CA, USA) and 0.5 mM glutamine (Life Technologies, Carlsbad, CA, USA). Cells were plated at a density of 2 × 10^5^ cells/cm^2^ onto culture dishes, which had been coated with poly-L-lysine (Biocoat, BD Biosciences, San Jose, CA, USA), and grown in the same medium in a humidified 5% CO_2_ incubator at 37°C. In the present study, OGD was used to induce ischemia. To initiate ischemia, we used the same incubator in combination with a Hypoxic Workstation (gas mixture of 0.1% O_2_, 94.9% N_2_ and 5% CO_2_, 37°C).

### Drug Application

Neurons were pre-exposed to OGD conditions for 1 h and subsequently treated with different concertrations of RSV (0.5, 5, or 50 μM) for 48 h. Cells not treated with RSV or OGD served as a negative control. To evaluate the effect of Notch1 on mitochondrial function and biogenesis, a potent and specific inhibitor of the Notch1 pathway, DAPT, N-[N-(3,5-difluorophenacetyl)-L-alanyl]-S-phenylglycinet-butyl ester, was added to the medium at 10 μM (in 0.1% dimethyl sulfoxide; Sigma, USA), 30 min before the RSV treatment.

### Neurite Outgrowth Assay

After 48 h of drug application, primary cortical neurons were fixed with 4% paraformaldehyde for 20 min and then processed for immunocytochemistry. Briefly, neurons were incubated with the mouse monoclonal anti-β-III-tubulin antibody (Tuj-1, 1:500, Sigma, USA) overnight at 4°C, followed by the donkey anti-mouse IgG, FITC-conjugated secondary antibody (1:200, CWBIO, China) for 1 h at 37°C. Stained cells were imaged with an upright fluorescence microscope (Olympus, Japan). The length of the longest neurite of a Tuj-1-positive cell and the total neurite length per cell were measured using ImageJ software. Approximately 60 Tuj-1-positive cells per condition were measured.

### Measurement of Mitochondrial Membrane Potential (MMP)

The Mitochondrial Membrane Potential (MMP) of cortical neurons in different conditions was measured by using the JC-1 assay kit (Beyotime, China), according to the manufacturer’s instructions. In brief, after the described treatments, neurons were collected and incubated with JC-1 staining solution (5 μg/mL) for 20 min at 37°C. Cells were then rinsed twice with JC-1 staining buffer and centrifuged at 600× *g* at 4°C for 15 min. The cells were resuspended with JC-1 staining buffer and the fluorescence intensity was detected using a monochromator microplate reader (Tecan, Switzerland). Fluorescence images were also obtained in green or red channels using an upright fluorescence microscope (Olympus, Japan). The fluorescence at 529 (green) and 590 (red) nm was measured using the monochromator microplate reader. The ratio of red to green fluorescence in different conditions was normalized to the respective one in the control condition, which was considered to have 100% MMP, and plotted graphically. Data were presented as percent of control.

### Measurement of ROS

Intracellular ROS levels were quantified with the ROS assay kit (Beyotime, China) as previously reported (He et al., 2017). In brief, cortical neurons were incubated with 10 μM 2,7-dichlorofluorescein diacetate (DCF-DA) for 1 h at 37°C in the dark and then resuspended in PBS. Intracellular ROS production indicated by the fluorescence intensity of the probe 2,7-dichlorodihydro-fluorescein diacetate (H_2_DCF-DA) was detected using a luminescence spectrometer with the excitation source set at 488 nm and the emission one at 525 nm. The values obtained at various conditions were expressed as the percentage change compared to the control condition.

### Detection of Cellular ATP Levels

The cellular ATP levels were determined using an ATP assay kit according to the manufacturer’s instructions (Beyotime, China). Luminesence was measured with a monochromatic microplate reader (Tecan, Switzerland). Data were presented as percentages compared to the control condition.

### Mitochondrial DNA (mtDNA) Quantification

Total DNA from cortical neurons was extracted using the DNeasy Blood and Tissue kit (Qiagen, Germantown, MD, USA) according to previous reports (Tian et al., 2017). Mitochondrial DNA (mtDNA) copy number was measured by real-time PCR using an ABI 7500 real-time PCR system (Applied Biosystems, Foster, CA, USA) with the SYBR Green detection method. The relative mtDNA copy number was determined by comparison to nuclear DNA (rRNA 18S). The primers for mtDNA were as follows: forward: 5′-AACACGA TCAGGCAACCAAA-3′, and reverse: 5′-GGTAGCGGGTGAGTTGTCAG-3′. The primers for rRNA 18S were: forward: 5′-GGACAGCGGGTGAGTTGTCA-3′, and reverse: 5′-ACCTTCGTTATCGGAATACC-3′.

### Quantitative Real-Time PCR

Quantitative real-time PCR (qRT-PCR) was performed according to previous reports (Dai et al., 2014; He et al., 2016). Briefly, total RNA was isolated from cortical neurons using Trizol reagent (Invitrogen, Carlsbad, CA, USA). Reverse transcription was carried out using the First-strand cDNA synthesis kit (Fermentas International Inc., Burlington, Canada) and the cDNA was amplified by a real-time PCR system (Applied Biosystems, Carlsbad, CA, USA) in the presence of a fluorescent dye (SYBR Green I, CWBIO). The relative abundance of specific mRNAs was calculated after normalization with the glyceraldehyde 3-phosphate dehydrogenase mRNA. The samples were tested in triplicates. Primers for all qRT-PCR experiments were listed as follows:

**Table:** 

NRF-1:	forward: 5′-GAGTGACCCAAACCGAACA-3′,
	reverse: 5′-GGAGTTGA GTATGTCCGAGT-3′;
TFAM:	forward: 5′-GGTGTATGAAGCGGATTT-3′,
	reverse: 5′-CTTTCTTCTTTAGGCGTTT-3′;
GAPDH:	forward: 5′-AAGGTGAAGGTCGGAGTCAA-3′,
	reverse: 5′-AATGAAGGGGTCATTGATGG-3′.

### Statistical Analysis

Statistical analysis was performed using SPSS version 16.0. All data were presented as mean ± SEM. One-way analysis of variance (ANOVA) was performed for comparisons among groups, and SNK-q test was used for *post hoc* multiple comparisons. *p < 0.05 was considered to be statistically significant.

## Results

### RSV Restores Neuritogenesis in Cortical Neurons Damaged by OGD

**Figure 1:**
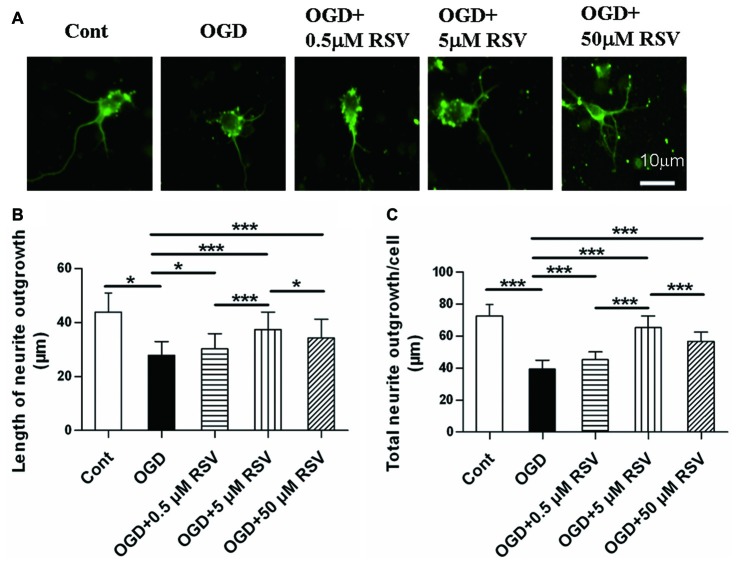
Effect of rosuvastatin (RSV) on neurite outgrowth in cultured cortical neurons. Neurons were pre-exposed to oxygen-glucose deprivation (OGD) for 1 h and subsequently treated with or without RSV for 48 h. Cells were stained with anti-β-III-Tubulin (Tuj-1) antibody. **(A)** Representative images of Tuj-1-positive neurons (green) in different conditions. Scale bar represents 10 μm. **(B)** Quantitative analysis of the length of the longest neurite. Results are presented as the mean ± SEM. *p < 0.05, ***p < 0.005, *n* = 60 per condition. **(C)** Quantitative analysis of the total neurite length per cell. Results are presented as the mean ± SEM. *p < 0.05, ***p < 0.005, *n* = 60 per condition.

First, we examined the effect of RSV on the neurite outgrowth of cortical neurons under OGD. As shown in Figures 1A,B, neurons under OGD showed a remarkable decrease in neurite outgrowth (27.86 ± 5.11 μm, *n* = 60), compared to control cells (43.84 ± 7.15 μm, *n* = 60, *p* < 0.005). On the other hand, the treatment with different concentrations of RSV (0.5, 5, or 50 μM), for a period of 48 h after OGD injury, resulted in a significant recovery of neurite outgrowth. Compared with the OGD-treated cells (27.86 ± 5.11 μm), the length of the longest neurite was 30.25 ± 5.60 μm (*n* = 60, *p* = 0.016), for neurons treated with 0.5 μM RSV, 37.36 ± 6.55 μm, for those treated with 5 μM RSV (*n* = 60, *p* < 0.005), and 34.24 ± 7.02 μm, for those treated with 50 μM RSV (*n* = 60, *p* < 0.005). The fold change in the total neurite length between the untreated OGD-exposed cells and the treated ones with 0.5, 5 and 50 μM of RSV was 1.15, 1.65 and 1.44, respectively (*n* = 60, *p* < 0.005, Figure 1C). These results indicated that RSV could effectively improve neuritogenesis in cortical cells, previously injured by OGD. We chose 5 μM RSV to carry out subsequent experiments, considering that this concentration demonstrated the highest potential in inducing neurite outgrowth compared with the one of 0.5 (*n* = 60, *p* < 0.005) or 50 μM (*n* = 60, *p* < 0.005).

### RSV Preserves Mitochondrial Function in Cortical Neurons under OGD

**Figure 2:**
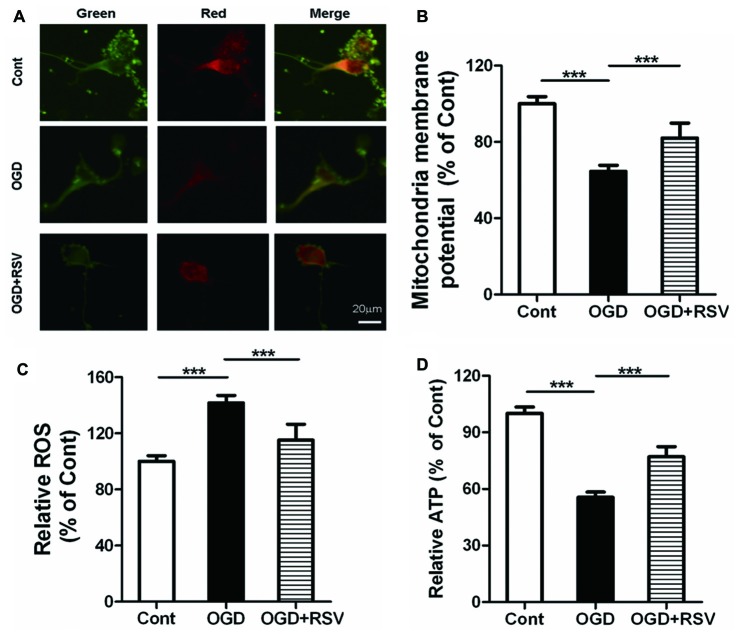
Effects of RSV on mitochondrial function and ATP levels in cortical neurons exposed to OGD. Cells were exposed to OGD and incubated with the indicated concentrations of RSV. **(A)** Mitochondrial membrane potential (MMP) as determined using the JC-1 assay. Scale bar represents 20 μm. **(B)** MMP as determined using the JC-1 assay kit in different conditions (control, OGD-exposed and OGD-exposed plus RSV). The results are expressed as the mean ± SEM. ***p < 0.005, *n* = 6 per group. **(C)** Measurement of reactive oxygen species (ROS) generation in different conditions (control, OGD-exposed, and OGD-exposed plus RSV). Results are expressed as the mean ± SEM. ***p < 0.005, *n* = 6 per group. **(D)** ATP production measured in different conditions (control, OGD-exposed and OGD-exposed plus RSV). Results are expressed as the mean ± SEM. ***p < 0.005, *n* = 6 per condition.

We next investigated the involvement of mitochondrial function in RSV-induced neurite outgrowth. Indicators of mitochondrial function were assessed in primary cortical neurons exposed to RSV under OGD conditions. Exposure of neurons to OGD for 1 h resulted in dissipation of the MMP (*n* = 6, *p* < 0.005) and an increase in ROS production (*n* = 6, *p* < 0.005). On the other hand, the treatment of cells with RSV, counteracted these effects. The MMP recovered (*n* = 6, *p* < 0.005; Figures 2A,B) and ROS accumulation was significantly reduced (*n* = 6, *p* < 0.005; Figure 2C), in comparison to untreated OGD-exposed cells. The above-mentioned results suggest that RSV can reverse the mitochondrial dysfunction, induced by OGD.

### RSV Elevates the Energy Metabolism of Primary Cultured Neurons Suppressed by OGD

As mitochondria are the main source of energy generation, we detected ATP levels, to assess the energy metabolism of primary cultured neurons. RSV significantly reversed the decrease in cellular ATP levels, which was observed following exposure to OGD (*n* = 6, *p* < 0.005; Figure 2D). These results indicate that RSV can increase the energy metabolism, impaired by OGD.

### RSV Promotes Mitochondrial Biogenesis after OGD Exposure

**Figure 3:**
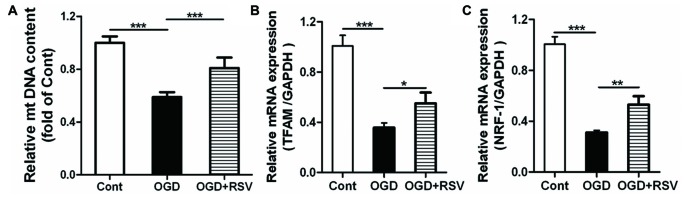
Effect of RSV on mitochondrial biogenesis in cortical neuron cultures. Cells were pre-exposed to OGD for 1 h and subsequently treated with or without RSV for 48 h. **(A)** Mitochondrial DNA (mtDNA) content measurements. Results are expressed as the mean ± SEM. *p < 0.05, **p < 0.01, ***p < 0.005, *n* = 6 per condition. **(B,C)** mRNA expression of mitochondrial transcription factor A (TFAM) **(B)** and nuclear respiratory factor 1 (NRF-1; **C**) as measured using quantitative real-time PCR. Data are shown as the mean ± SEM. *p < 0.05, **p < 0.01, ***p < 0.005, *n* = 6 per condition.

To determine whether the altered mitochondrial function and energy metabolism are related to mitochondrial biogenesis, we also estimated the mtDNA content in different conditions (Figure 3A). The results showed that OGD exposure significantly decreased mtDNA content, whereas RSV treatment abrogated this effect (*n* = 6, *p* < 0.005; Figure 3A).

Mitochondrial transcription factor A (TFAM) and nuclear respiratory factor 1 (NRF-1) are major regulators of mitochondrial biogenesis. Therefore, we measured the expression of these factors, using qRT-PCR (Figures 3B,C). The results showed that RSV treatment after OGD exposure significantly increased the mRNA expression of TFAM and NRF-1 (*n* = 6, *p* < 0.05; Figures 3B,C).

### Notch1 Mediates the Protective Effect of RSV on Mitochondrial Function and Biogenesis in Cortical Neurons Exposed to OGD

**Figure 4:**
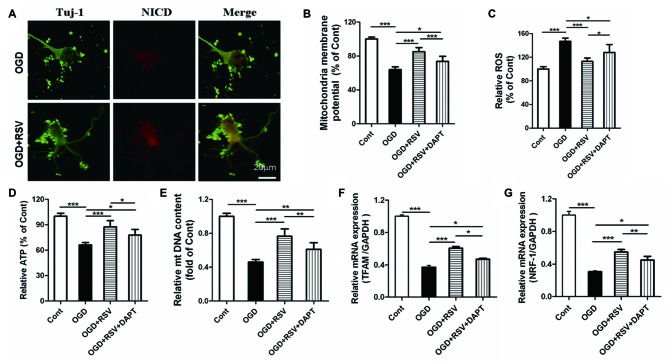
Notch1 pathway is involved in the beneficial effects of RSV on neuronal function and mitochondrial biogenesis under OGD conditions. **(A)** Double immunofluorescence staining for anti-Notch intracellular domain (NICD) and anti-β-III-Tubulin (Tuj-1) antibodies. NICD (red), a marker for activation of Notch1 pathway, is expressed in Tuj-1-positive neurons (green) and its expression is increased in OGD-exposed cells treated with RSV compared to non-treated cells. **(B–D)** Effect of DAPT, a specific inhibitor of the Notch1 pathway, on the RSV-induced improvement of mitochondrial function as assessed by measuring MMP **(B)**, ROS levels **(C)**, and ATP levels **(D)**. Data are presented as mean ± SEM. *p < 0.05, **p < 0.01, ***p < 0.005, *n* = 6 per condition. **(E–G)** Effect of DAPT on RSV-induced mitochondrial biogenesis as assessed by measuring mtDNA **(E)** and the mRNA levels of TFAM **(F)** and NRF-1 **(G)**. Data are presented as mean ± SEM. *p < 0.05, **p < 0.01, ***p < 0.005, *n* = 6 per condition.

Based on our finding that RSV preserved the mitochondrial function in primary cultured neurons exposed to OGD, we explored whether Notch1 was involved in this process. The release of the Notch intracellular domain (NICD) is widely used as a marker for Notch1 pathway activation. In our study, we examined NICD expression via immunocytochemical analysis. We found that NICD was expressed in cortical neurons, exposed to OGD, which suggested that endogenous Notch1 might be activated upon neuronal injury and mediate OGD remodeling. In these conditions, we found that RSV treatment increased the expression of NICD (Figure 4A; data not shown). Next, we used the Notch1 pathway inhibitor DAPT to assess mitochondrial function. Compared to the RSV-treated OGD-exposed cells, DAPT partially reduced MMP (*n* = 6, *p* < 0.005; Figure 4B), boosted ROS production (*n* = 6, *p* < 0.05; Figure 4C), and decreased ATP levels (*n* = 6, *p* < 0.05; Figure 4D). Therefore, inhibition of the Notch1 pathway reverts the effects of RSV, at least to a certain extent. We also explored whether DAPT could affect mitochondrial biogenesis in primary cultured neurons exposed to OGD. Compared to the RSV-treated cells, DAPT partialy decreased the mtDNA content (*n* = 6, *p* < 0.01; Figure 4E) and reduced the mRNA levels of TFAM (*n* = 6, *p* < 0.05; Figure 4F) and NRF-1 (*n* = 6, *p* < 0.01; Figure 4G).

Additional, we did the following experiments whether DAPT itself had detrimental effects. The length of the longest neurite, total neurite outgrowth per cell, MMP, ROS, ATP, mtDNA, TFAM and NRF-1 were detected. The data were shown in the supplementary data. The results showed that there were no signifcant diferences in the length of the longest neurite, total neurite outgrowth per cell, MMP, ROS, ATP, mtDNA, TFAM and NRF-1 between OGD+DAPT and OGD group (see Supplementary Figures S1–S3 in Supplementary Data), suggesting that DAPT itself has no detrimental effects.

## Discussion

In this present study, we demonstrated that RSV restores neuritogenesis in primary cortical cells damaged by OGD. This action is possibly mediated by the improvement in mitochondrial function and biogenesis. Furthermore, we also elucidated that Notch1 is crucial for these RSV-dependent effects. To the best of our knowledge, this is the first report to provide evidence for the effect of RSV on cortical neuritogenesis following OGD and the involvement of Notch1 in RSV-induced mitochondrial biogenesis and functional improvement.

Statins act as inhibitors of the HMG-CoA reductase and have been extensively used for the treatment of hypercholesterolemia (Kahveci et al., 2014). Numerous studies have reported that statins may have neuroprotective properties, as demonstrated by the reduction of the affected region following focal cerebral ischemia and the protection of cortical neurons from excitotoxicity (Asahi et al., 2005; Bösel et al., 2005). Recent studies have shown that treatment of cortical neurons, cultured under OGD/reoxygenation conditions, with RSV, a novel HMG-CoA reductase inhibitor, was neuroprotective for the cells (Savoia et al., 2011). RSV exerts considerable protective effects on neural tissue against oxidative damage after spinal cord ischemia/reperfusion injury, improves cognitive functions in rats with diazepam-induced amnesia, and preserves long-term memory (Yavuz et al., 2013). Our results demonstrated that RSV, at a concentration of 5 μM, shows the maximum effects on enhancing neuritogenesis in cortical neurons exposed to OGD.

Neurite outgrowth is an energy-consuming process that primarily depends on mitochondria. Mitochondria may play a crucial role in controlling neuroplasticity, including neurite extension (Mattson, 2007; Cheng et al., 2010). It has been reported that modulating mitochondrial function may impact neurite outgrowth (Habash et al., 2015). Furthermore, impaired mitochondrial function may disturb neuroplasticity following stroke (Cheng et al., 2010). Additionally, mitochondria may influnce the generation of ROS (Onyango et al., 2011), whose accumulation, caused by ischemia, could disrupt MMP and funcion. Damaged mitochondria can, in turn, generate more ROS (Bai et al., 2017). In this study, we found that the effects of RSV on neurite outgrowth correlated with improved mitochondrial function, as indicated by elevated MMP and ATP levels, as well as decreased ROS generation. These results suggest that mitochondrial function may be, at least in part, involved in RSV-induced neurite outgrowth.

Mitochondrial biogenesis is a highly regulated process, which occurs continuously in healthy cells and is crucial for cellular adaptation (Nikoletopoulou and Tavernarakis, 2014). Recent evidence has suggested a subtle link between mitochondrial biogenesis and neurological disorders (Mandemakers et al., 2007). Mitochondrial biogenesis has been found to counteract the detrimental effects of oxidative stress and has been suggested as a novel target of the repair mechanism (Cheng et al., 2010; Habash et al., 2015). *In vitro* studies have also suggested that impaired biogenesis contributes to the reduction of mitochondrial function after cerebral ischemia (Wang et al., 2014); however, its enhancement may reduce ischemic brain injury (Valerio et al., 2011). Although previous studies have indicated that RSV might impair mitochondrial function and biogenesis (Broniarek and Jarmuszkiewicz, 2016), our results show that RSV treatment restores the OGD-induced mtDNA loss in cortical neurons. TFAM and NRF-1 play an important role in the initiation of mtDNA replication and the transcription of mitochondrial encoded genes (Campbell et al., 2012). Therefore, we measured the mRNA levels of TFAM and NRF-1 in neurons exposed to OGD and treated with or without RSV. Our results revealed that RSV treatment significantly increased the mRNA levels of these factors. Collectively, our findings indicate that the RSV-induced neurite outgrowth against OGD exposure can be partially explained by the improved mitochondrial function and their enhanced biogenesis. Recent studies have provided convincing evidence that RSV exerts its protective effect by decreasing ROS levels, inhibiting the opening of the mitochondrial permeability transition pore, and promoting mitochondrial biogenesis (Corsetti et al., 2014; Liu et al., 2017). Nonetheless, further studies will be required to determine the exact mechanism of RSV effects on mitochondria.

The Notch pathway plays a vital role in the regulation of cell proliferation, self-renewal and differentiation, and is involved in several disorders of the central nervous system (Lundqvist et al., 2013). A previous study suggested that this pathway could regulate neurite outgrowth (Sestan et al., 1999; Levy et al., 2002). It also has been reported that Notch1 influences neurite morphology, and can activate its native signal transduction pathway in postmitotic neurons. Beyond neurogenesis, Notch1 plays a physiologically vital role in the central nervous system (Berezovska et al., 1999). In two recent studies, using M1 macrophages or cell lines *in vitro*, the Notch1 pathway was shown to enhance mtDNA transcription, ATP levels, and mitochondrial function (Basak et al., 2014; Xu et al., 2015). In addition, two statins, namely atorvastatin and simvastatin, have been shown to exert their effects, following stroke, through Notch signaling; the first, by increasing cell proliferation in the subventricular zone (Chen et al., 2008) and the second, by promoting arteriogenesis (Zacharek et al., 2009). Thus, we hypothesized that the Notch1 pathway was implicated in the regulation of RSV-induced mitochondrial biogenesis and function in cortical neurons. Our results show that, under OGD conditions, Notch1 signaling is active in primary cortical neurons and that its inhibition reverses the positive effects of RSV treatment on mitochondrial biogenesis and function. These results suggest that this pathway may, at least partially, contribute to the RSV-induced mitochondrial function and biogenesis in cortical neurons *in vitro*, which may represent a potent therapeutic strategy to promote brain plasticity after ischemic injury.

## Conclusion

The present study has demonstrated that RSV promotes neurite outgrowth in primary cortical neurons, thus shielding them against OGD. RSV seems to be vital in preserving mitochondrial function and improving mitochondrial biogenesis and these effects are, at least in part, mediated by the Notch1 pathway. These findings highlight Notch1 signaling and mitochondria as important players and novel therapeutic targets in promoting brain plasticity.

## Author Contributions

WH and XT: study concept design and drafting of the manuscript. WH: collection of data. YL and XT: analysis and interpretation of data. All authors approved the final version of the manuscript.

## Conflict of Interest Statement

The authors declare that the research was conducted in the absence of any commercial or financial relationships that could be construed as a potential conflict of interest.

## Supplementary Material

FIGURE S1 Effect of DAPT-only treatment on neurite outgrowth in primary cultured cortical neurons under oxygen-glucose deprivation (OGD). **(A)** Quantitative data of the longest neurite length of cultured cortical neurons. Results were presented as mean ± SEM. ns, non-significant, *n* = 60 per condition. **(B)** Quantitative data of total neurite length per cell. Results were presented as mean ± SEM. ns, non-significant, *n* = 60 per condition.

FIGURE S2 Effect of DAPT-only treatment on mitochondrial function and ATP levels in cortical neurons exposed to OGD. **(A)** Mitochondrial membrane potential (MMP) was determined using JC-1 assay kit in different groups (OGD group, and DAPT group). Results were expressed as the mean ± SEM. ns, non-significant, *n* = 6 per condition. **(B)** The generation of ROS in different groups (OGD group and DAPT group). Results were expressed as the mean ± SEM. ns, non-significant, *n* = 6 per condition. **(C)** The ATP levels were measured in different groups (OGD group, and DAPT group). Results were expressed as the mean ± SEM. ns, non-significant, *n* = 6 per condition.

FIGURE S3 Effect of DAPT-only treatment on mitochondrial biogenesis in cortical neurons exposed to OGD. **(A)** The mitochondrial DNA (mtDNA) content was measured. Results were expressed as the mean ± SEM. ns, non-significant, *n* = 6 per group. The expression of mitochondrial transcription factor A (TFAM; **B**), and nuclear respiratory factor 1 (NRF-1; **C**) at mRNA levels was measured by Quantitative Real-Time PCR. Data are shown as mean ± SEM. ns, non-significant, *n* = 6 per condition.
