# Supplementary figures and images for: Rosuvastatin Improves Neurite Outgrowth of Cortical Neurons against Oxygen-Glucose Deprivation via Notch1-mediated Mitochondrial Biogenesis and Functional Improvement
Source: Front Cell Neurosci. 2018 Jan 17;12:6. doi: 10.3389/fncel.2018.00006 (PMC5776084; doi:10.3389/fncel.2018.00006)

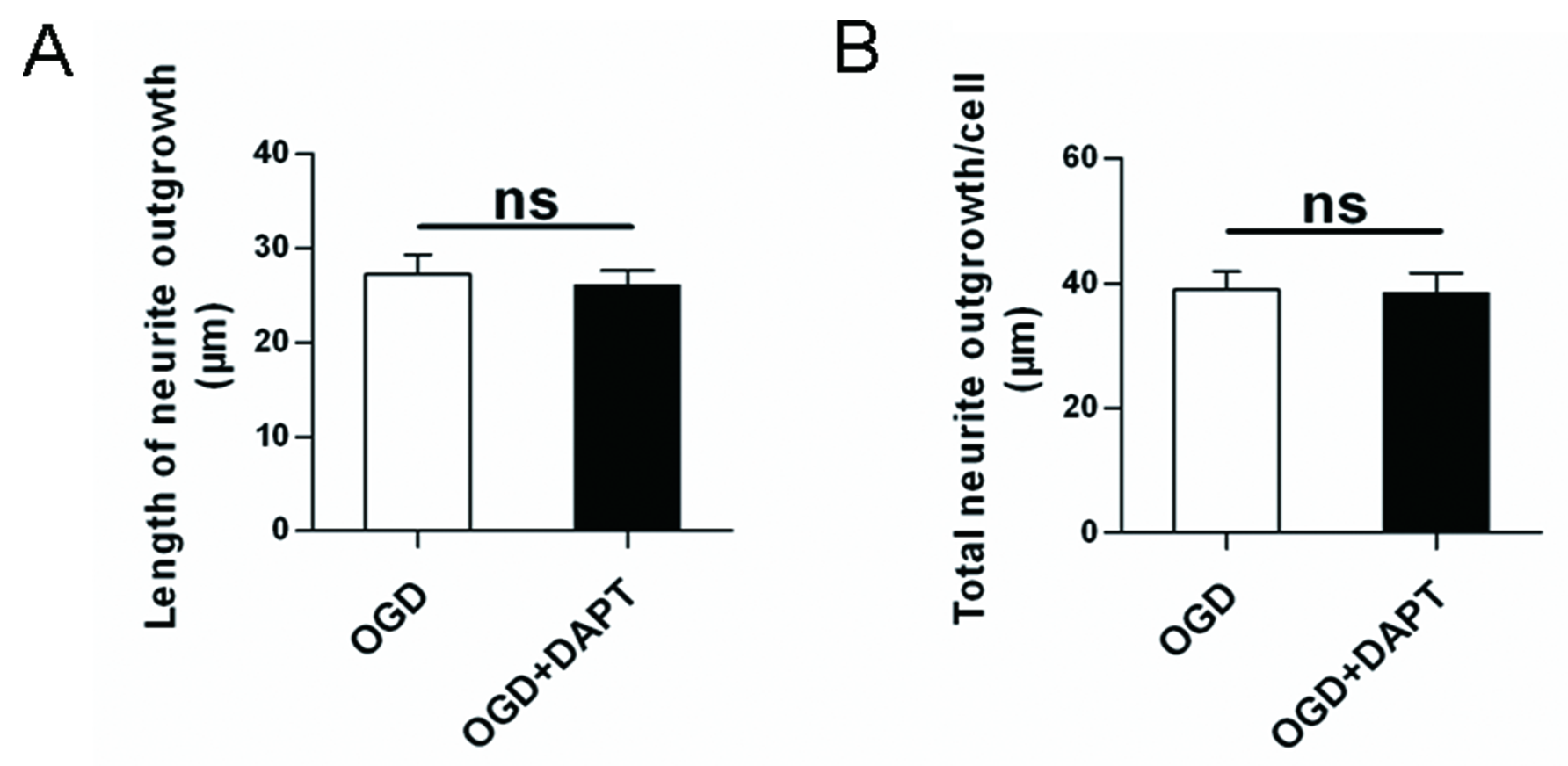

Supplement: FIGURE S1 — Effect of DAPT-only treatment on neurite outgrowth in primary cultured cortical neurons under oxygen-glucose deprivation (OGD). (A) Quantitative data of the longest neurite length of cultured cortical neurons. Results were presented as mean ± SEM. ns, non-significant, n = 60 per condition. (B) Quantitative data of total neurite length per cell. Results were presented as mean ± SEM. ns, non-significant, n = 60 per condition. [file Image_1.tif]

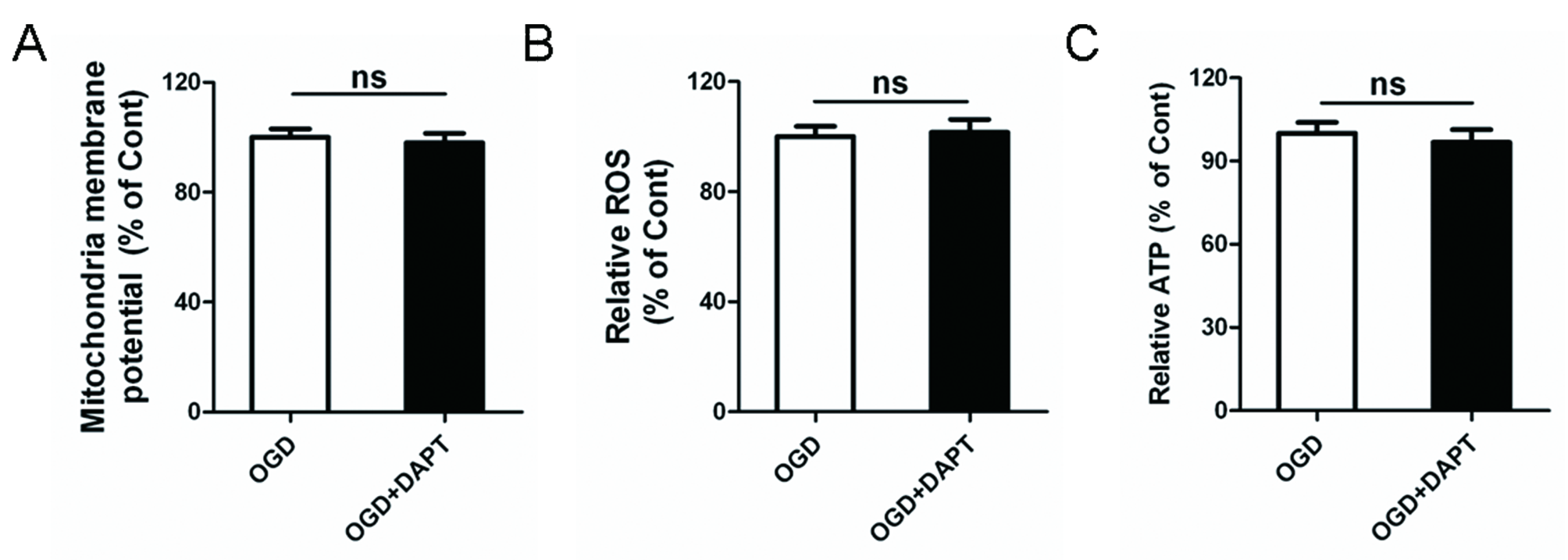

Supplement: FIGURE S2 — Effect of DAPT-only treatment on mitochondrial function and ATP levels in cortical neurons exposed to OGD. (A) Mitochondrial membrane potential (MMP) was determined using JC-1 assay kit in different groups (OGD group, and DAPT group). Results were expressed as the mean ± SEM. ns, non-significant, n = 6 per condition. (B) The generation of ROS in different groups (OGD group and DAPT group). Results were expressed as the mean ± SEM. ns, non-significant, n = 6 per condition. (C) The ATP levels were measured in different groups (OGD group, and DAPT group). Results were expressed as the mean ± SEM. ns, non-significant, n = 6 per condition. [file Image_2.tif]

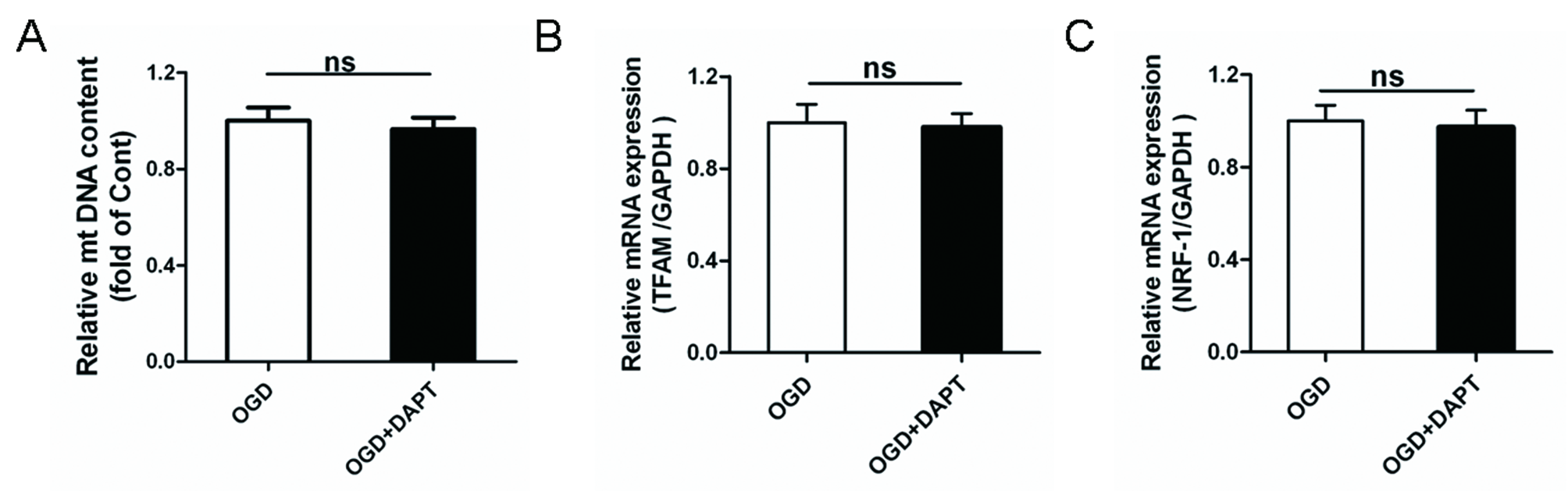

Supplement: FIGURE S3 — Effect of DAPT-only treatment on mitochondrial biogenesis in cortical neurons exposed to OGD. (A) The mitochondrial DNA (mtDNA) content was measured. Results were expressed as the mean ± SEM. ns, non-significant, n = 6 per group. The expression of mitochondrial transcription factor A (TFAM; B), and nuclear respiratory factor 1 (NRF-1; C) at mRNA levels was measured by Quantitative Real-Time PCR. Data are shown as mean ± SEM. ns, non-significant, n = 6 per condition. [file Image_3.tif]
